# Nanomaterials for Autophagy-Related miRNA-34a Delivery in Cancer Treatment

**DOI:** 10.3389/fphar.2020.01141

**Published:** 2020-07-24

**Authors:** Priyanka Sharma, Ilaria Dando, Raffaele Strippoli, Suresh Kumar, Alvaro Somoza, Marco Cordani, Marco Tafani

**Affiliations:** ^1^ Department of Pathology, University of New Mexico Health Sciences Center, Albuquerque, NM, United States; ^2^ Section of Biochemistry, Department of Neurosciences, Biomedicine and Movement Sciences, University of Verona, Verona, Italy; ^3^ Department of Molecular Medicine, Sapienza University of Rome, Rome, Italy; ^4^ Gene Expression Laboratory, National Institute for Infectious Diseases “Lazzaro Spallanzani” IRCCS, Rome, Italy; ^5^ Department of Molecular Genetics and Microbiology, University of New Mexico Health Sciences Center, Albuquerque, NM, United States; ^6^ IMDEA Nanociencia, Madrid, Spain; ^7^ Department of Experimental Medicine, Sapienza University of Rome, Rome, Italy

**Keywords:** miRNA-34a, miRNAs, autophagy, cancer treatment, nanoparticles

## Abstract

Autophagy is an evolutionary conserved physiological process with a fundamental role during development, differentiation, and survival of eukaryotic cells. On the other hand, autophagy dysregulation is observed in many pathological conditions, including cancer. In particular, tumor growth and progression are accompanied and promoted by increased autophagy that allows cancer cells to escape apoptosis and to proliferate also in harsh microenvironments. It is, therefore, clear that the impairment of the autophagic process may represent a valid strategy to inhibit or reduce cancer growth and progression. Among the plethora of molecular players controlling cancer growth, a group of small endogenous noncoding RNAs called microRNAs (miRNAs) has recently emerged. In fact, miRNAs can act as either oncogenes or oncosuppressors depending on their target genes. Moreover, among miRNAs, miRNA-34a has been connected with both tumor repression and autophagy regulation, and its expression is frequently lost in many cancers. Therefore, enforced expression of miRNA-34a in cancer cells may represent a valid strategy to reduce cancer growth. However, such strategy is limited by the fast biodegradation and short half-life of miRNA-34a and by the lack of an efficient intracellular delivery system. The following review describes the autophagic process and its role in cancer as well as the role of miRNAs in general and miRNA-34a in particular in regulating tumor growth by modulating autophagy. Finally, we describe the use of nanoparticles as a promising strategy to selectively deliver miRNA-34a to tumor cells for therapeutic and diagnostic purposes.

## Introduction

Autophagy is an intracellular process with a role in several pathological conditions including cancer, neurodegenerative disorders, and infectious diseases (Mizushima et al., 2011; Deretic et al., 2013; Dikic and Elazar, 2018), all processes in which cytosolic wastes are delivered to the lysosomes for degradation and recycling (Mizushima et al., 2011). Recently, Yoshinori Ohsumi was awarded the Nobel prize for his studies on the basic mechanisms of autophagy in yeast (Tooze and Dikic, 2016). However, the understanding of the molecular mechanisms controlling autophagy induction and modulation in the physiopathology of mammalian cells is still far from being elucidated.

MiRNAs are endogenous small noncoding RNAs that participate in the regulation of gene expression, whose dysregulation is mechanistically implicated in many pathological processes, including autophagy and tumorigenesis (Wang et al., 2013; Peng and Croce, 2016). Among the plethora of miRNAs analyzed, miRNA-34a expression is abnormally regulated in many cellular processes, including cell division, senescence, apoptosis, autophagy, and tumorigenesis (Huang et al., 2014). Hence, since the two latter processes are tightly connected, the re-establishment of endogenous intracellular levels of miRNAs and, especially, of miRNA-34a may block pathologic autophagy and counteract cancer progression. However, the low stability of miRNAs and their difficult delivery into cells restrict their application in clinical practice (Chen et al., 2015).

In this sense, advances in nanotechnology have led to the development of many different nanostructures, which can be employed as drug delivery systems to improve the properties of a variety of bioactive compounds, including nucleic acids (Mei et al., 2019). For these reasons, nanocarriers are being explored to convey therapeutic miRNAs in order to interfere with key biological processes involved in tumor progression, including autophagy.

In this review, we summarize recent studies describing the use of nanoparticles for the delivery of miRNA-34a in cancer cells, alone or in combination with traditional chemotherapy drugs for the inhibition of different oncogenic pathways, with emphasis on autophagy regulation. Hence, the delivery of miRNA-34a combined to nanostructures may improve its biodistribution and accumulation at the target site, with some papers showing encouraging results, both in *in vitro* and *in vivo* studies.

## Autophagy: When the Cells Eat Themselves

Autophagy is an evolutionarily conserved cellular homeostatic process that consists in the formation of double-membrane structures that engulf cytosolic wastes, including damaged organelles, protein aggregates, or invading pathogens, and fuse with lysosomes to degrade their content (Mizushima et al., 2011). Autophagy is further subdivided into several categories depending on its site of action, for instance, when it takes place in pathogens is called xenophagy (Levine, 2005), mitophagy in the mitochondria (Narendra et al., 2008), pexophagy in the peroxisomes (Nazarko et al., 2007), ERphagy in the endoplasmic reticulum (Chino et al., 2019. Khaminets et al., 2015), or lysophagy in the lysosomes (Maejima et al., 2013). Other relatively less studied forms of autophagy, called chaperon mediated autophagy involve molecular chaperones, such as HSP70 (Kaushik et al., 2006). Chaperone mediated autophagy does not require the whole autophagy machinery but involves the recruitment of the lysosomal membrane protein LAMP2 to substrate such as protein aggregates, damaged organelles and invading pathogens through chaperons and ultimately the degradation of the cargo (Kaushik et al., 2006). Chaperon mediated autophagy has several medical implications, including a role in cancer (Kaushik et al., 2011). Microautophagy is another form of autophagy that is mediated by the direct engulfment of cytoplasm and its components by membrane invaginations into lysosomes. A role of microautophagy has been demonstrated in various pathologies (Li et al., 2012). Another form of autophagy called LC3 dependent phagocytosis (LAP) has also been reported (Sanjuan et al., 2007). Unlike conventional autophagy, LAP involves the formation of a single membrane structure positive for autophagy marker LC3 around the phagocytic substrate and ultimately its fusion with the lysosome. Like conventional autophagy, LAP requires autophagy conjugation machinery (discussed in the next section) but does not require components of autophagy initiation machinery (Martinez et al., 2015). Another major difference between LAP and conventional autophagy is that LAP requires RUBICON (Run domain Beclin-1 interacting and cysteine-rich containing protein), which is an inhibitor of the conventional autophagy (Martinez et al., 2015). Like all other forms of autophagy, the role of LAP has been studied in various pathologies, such as inflammation related disorders (Martinez et al., 2016), neurodegeneration (Heckmann et al., 2019), and cancer (Cunha et al., 2018).

### Role of Autophagy in Cancer

**Figure 1:**
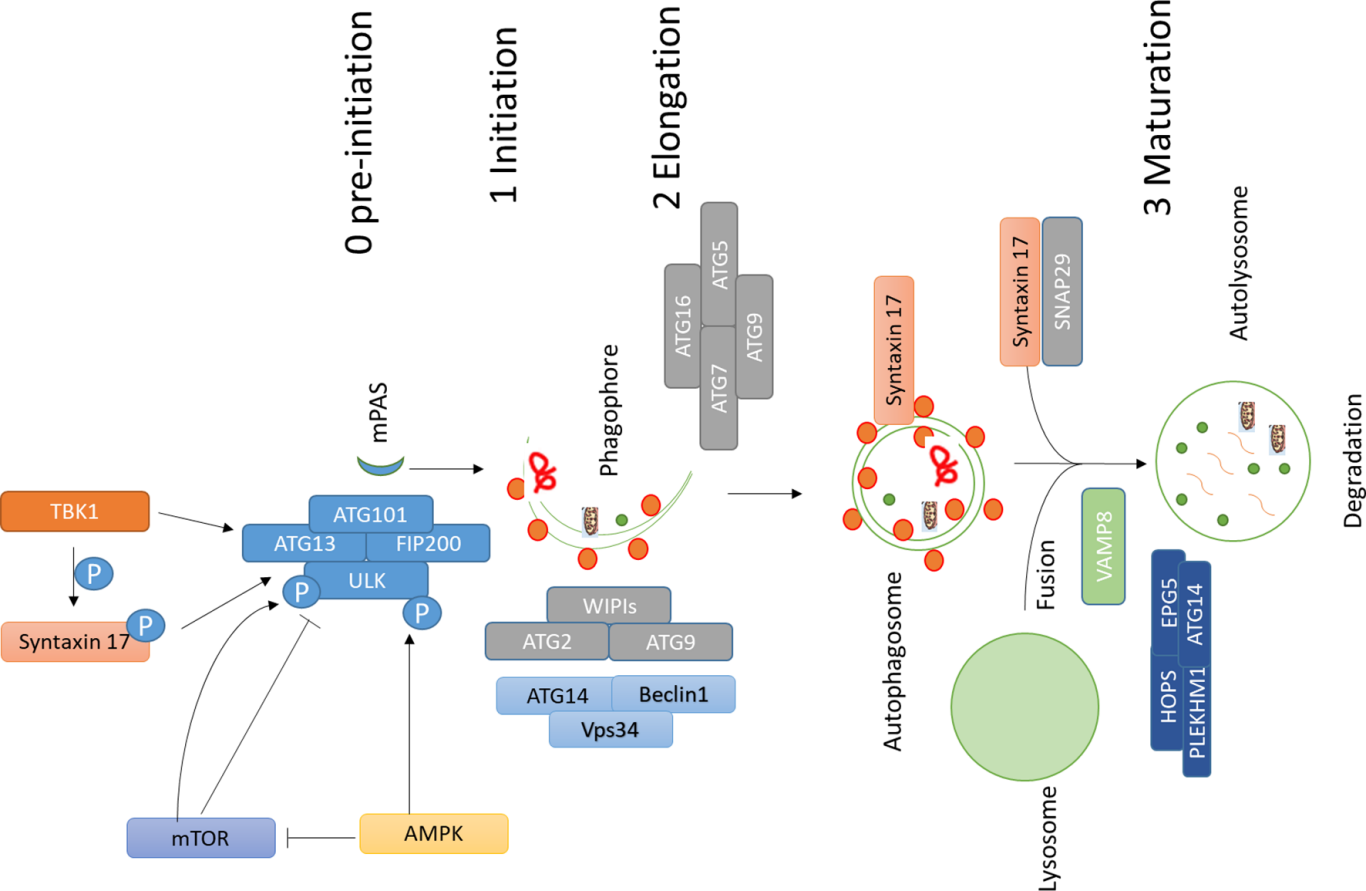
Main machinery and steps involved in autophagy. Autophagy involves different steps; assembly of ULK1 complex, which is negatively regulated by mTOR, which phosphorylates ULK1 and inhibits autophagy; AMPK phosphorylates ULK1 and activates autophagy initiation, AMPK might also affect this process by inhibiting mTOR activity by phosphorylating RAPTOR and TSC2. Autophagy initiation and assembly of ULK1 complex are controlled by TBK1 and its substrate Stx17. TBK1 and Stx17 also affect the formation of mPAS, which is then elongated to a cup-like phagophore by the action of WIPIs, ATG2, ATG9, and PI3K complex which comprises of VPS34, ATG14, and Beclin1. ATG conjugation machinery also helps in elongation and lipidation of the membrane. Once autophagosome is completed SNARE protein Stx17 gets recruited to autophagosomes and works together with SNAP29 and VAMP8 to regulate fusion between autophagosomes and lysosomes. Additional factors like HOPS, PLEKHM1, EPG5, and ATG14 help Stx17 in this process. Abbreviations: ATG, Autophagy related; AMPK, 5′ AMP-activated protein kinase; TBK1, TANK Binding Kinase 1; mTOR, mammalian Target of Rapamycin; SNARE, soluble NSF (N-ethylmaleimide-sensitive factor) attachment protein receptors).

The role of autophagy in cancer is somewhat controversial. Earliest reports suggested a role of autophagy against tumorigenesis (Liang et al., 1999) and afterwards, several reports found a role of autophagy favoring cell death of tumor cells in an apoptotic-dependent or independent way (Levine and Yuan, 2005; Pattingre et al., 2005; Kumar et al., 2013; Sierra et al., 2015). On the other hand, it was demonstrated that inhibition of autophagy could also hamper the antitumor T cell response, which is required for immunogenic cell death (Townsend et al., 2012; Ma et al., 2013). However, the observation that in a different setting the inhibition of autophagy does not suppress the immune response suggests the existence of autophagy-independent mechanisms of the immune response (Starobinets et al., 2016). Therefore, the role of autophagy in immunogenic cell death in cancer may vary from one condition to other, and no conclusion can be drawn based on the present literature. All these studies suggest that autophagy plays a dual role in cancer: its induction may protect against cancer at earlier stages of tumorigenesis, whereas at later stages cancer cells may utilize this process to escape from apoptosis and to promote uncontrolled growth, as well as resistance to stress”. However, to better clarify the proproliferative role of autophagy in cancer, many efforts have been made by using pharmacological modulators for cancer therapy (Amaravadi et al., 2007; Levy et al., 2017; Amaravadi et al., 2019). For instance, autophagy inhibitors chloroquine (CHQ) and hydroxychloroquine (HCQ) are being tested against different types of cancer (Levy et al., 2017; Amaravadi et al., 2019). Both CHQ and HCQ inhibit the fusion between autophagosomes and lysosomes occurring at the final stage of the autophagic process (Klionsky et al., 2016). Moreover, proteins active in different stages of autophagy are being analyzed as potential therapeutic targets in cancer. Autophagy is initiated by the ULK1 complex, and inhibition of ULK1 has shown encouraging results in cancer therapy (Egan et al., 2015; Martin et al., 2018). Likewise, suppression of FIP200, another component of autophagy initiation complex, also induces apoptosis, and inhibition of FIP200 limits early tumorigenesis and also the progression of breast cancer in a mouse model (Wei et al., 2011; Xia et al., 2017). Inhibition of VPS34 has also shown promising results (Dowdle et al., 2014; Ronan et al., 2014). ATG4B, which is required for the processing of mAtg8s and consequently, for the progression of autophagy, is also being targeted to inhibit cancer progression (Levy et al., 2017). Metabolism is one of the targets in cancer therapy. Autophagy also plays an important role in maintaining cancer cell metabolism (Rabinowitz and White, 2010; Guo et al., 2011). More recently, it was shown that autophagy is required for maintaining high circulating arginine levels necessary to promote cancer progression (Poillet-Perez et al., 2018). Also, proteins with multiple roles in autophagy could be targeted. An example is Stx17, which acts in different steps of autophagy: it regulates the formation of earliest autophagosomal structures (Kumar et al., 2019) and consequently the initiation of autophagy (Hamasaki et al., 2013; Sugo et al., 2018; Kumar et al., 2019). Moreover, Stx17 is a key regulator of fusion between autophagosomes and lysosomes (Diao et al., 2015), and this step is being targeted in various cancer using chloroquine and hydroxychloroquine (Levy et al., 2017). All these reports put forward the role of autophagy regulators like Stx17 as a potential new target in cancer therapy. **Figure 1**.

## miRNAs in Cancer

MicroRNAs (miRNAs) are a class of small endogenous noncoding RNAs of about 21–25 nucleotides that modulate gene expression by binding to the 3′-UTR region of a specific mRNA target. The main effect of the miRNA binding is the decreased expression of specific RNA sequences with consequences on several biological processes, including cell proliferation, differentiation, apoptosis, development, and tumorigenesis (Esquela-Kerscher and Slack, 2006). Indeed, the alteration of miRNA expression pattern has been shown to support cancer initiation, progression and dissemination (Berindan-Neagoe and Calin, 2014). Increasing evidences have shown that, depending on their target genes, miRNAs can sustain tumor, by acting as either oncogene or tumor suppressor, thus favoring evasion from growth suppressors, support of proliferation, resistance to cell death, angiogenesis, and activation of invasion and metastasis (Peng and Croce, 2016). In many cancer types, miRNA profile is abnormal due to gene amplifications or deletions, alteration of their transcriptional control, epigenetic dysregulations and defects in the miRNA biogenesis machinery. In particular, miRNA-34a, a tumor-suppressor miRNA that belongs to an evolutionarily conserved family of miRNAs, is deregulated in many human tumor types, including breast, lung, prostate cancer, cholangiocarcinoma, acute myeloid leukemia (AML), and multiple myeloma (MM). MiRNA-34a plays a key role in many cellular processes, including the regulation of cell division, senescence, apoptosis, and proliferation (Huang et al., 2014). Indeed, in prostate cancer, one of the most frequently diagnosed tumors, it has been shown that, among the 50 altered miRNAs that act in carcinogenesis, miR-34a expression is strongly downregulated (Liao et al., 2016). MiRNA-34a decreased expression may lead to a reduction in cell cycle arrest and apoptosis and to the enhancement of chemoresistance and autophagy. Also, in cholangiocarcinoma miR-34a downregulation is implicated in drug resistance and in the control of epithelial–mesenchymal transition (EMT) through an effect on Smad4 (Sun et al., 2017). Interestingly, the enforced expression of miR-34a in MM cells or its delivery in the tumor xenografts by direct intratumor or intravenous injection mimics the effect of other anticancer agents without producing toxicity, thus representing a new therapeutic strategy (Di Martino et al., 2014). Thus, all this evidence supports the analysis in tumors of miRNAs, and in particular of miRNA-34a, as well as the artificial restoration of their physiological levels for both diagnostic and therapeutic purposes.

### miRNA-34a in Autophagy Regulation

**Figure 2:**
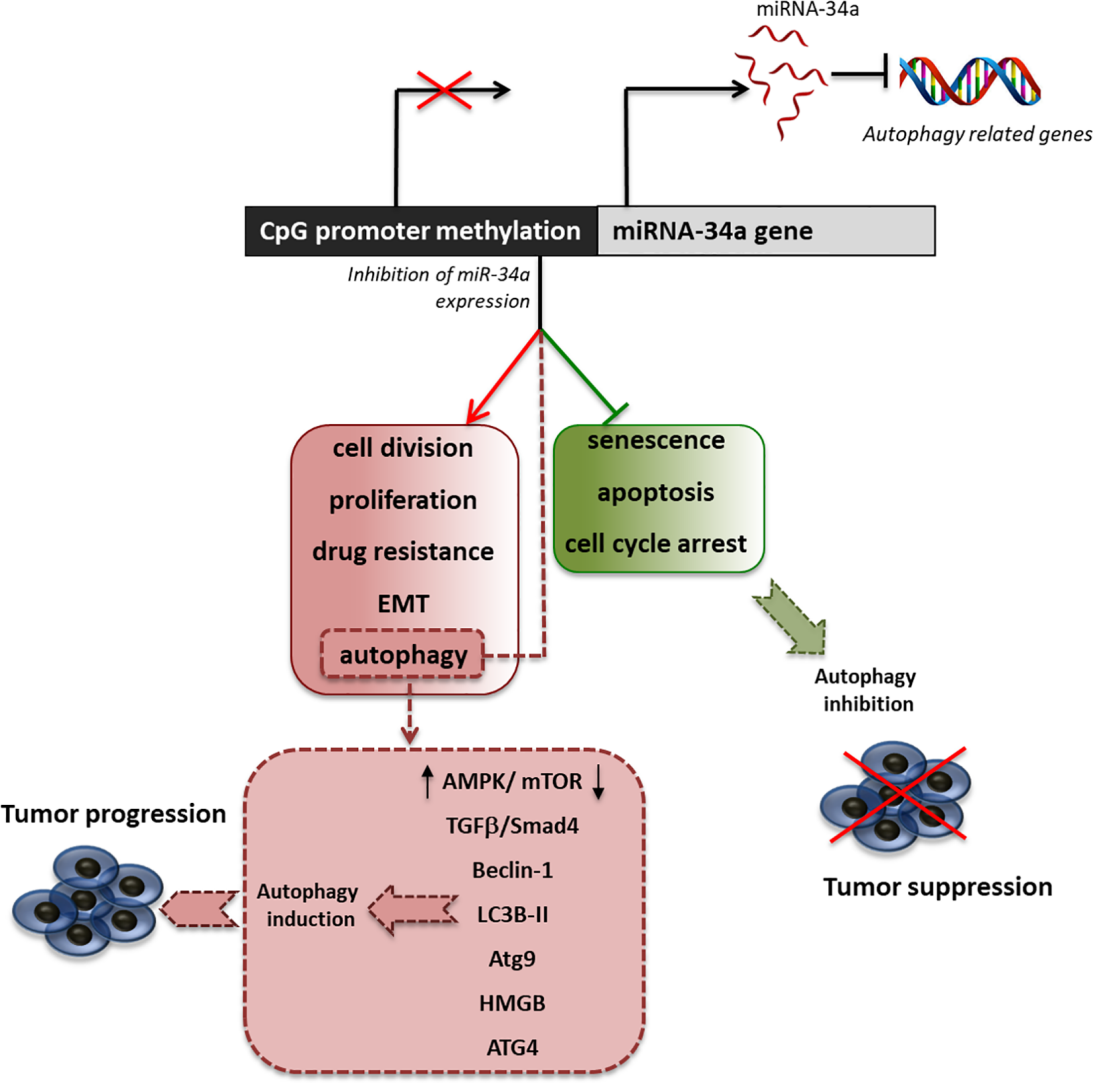
Effect of miRNA34a inhibition on autophagy. Schematic representation of promoter methylation, which represents the most common cause of miRNA34a decreased expression in tumors and of the principal effects of miRNA34a low expression in the sustainment of cancer proliferation. Particularly, we highlighted the main pathway involved in the regulation of autophagy by miRNA-34a.

Many studies have investigated the multiple effects associated with the decreased expression of miRNA-34a in support of tumor growth, showing that this miRNA is also an important regulator of autophagy (**Figure 2**). Indeed, Liao et al. demonstrated that in prostate cancer, miRNA-34a expression is decreased due to gene hypermethylation, correlating with higher cell proliferation, apoptosis abrogation, enhanced chemoresistance, and autophagy induction (Liao et al., 2016). By overexpressing miR-34a, they showed that the autophagy-related proteins, ATG4B, Beclin-1, and LC3B II/I, were downregulated. This led to enhanced chemosensitivity to the drugs doxorubicin and topotecan. Furthermore, they showed that the downregulation of miRNA-34a expression parallels the upregulation of ATG4B-induced autophagy through the AMPK/mTOR pathway regulation (Liao et al., 2016). Another study on colorectal cancer showed that the treatment with oxaliplatin decreased the level of miRNA-34a, with the consequent increase of drug resistance due to the activation of autophagy. This activation has been shown to be mediated by the regulation of the TGF-β/Smad4 pathway. Indeed, 34a in colorectal cancer patients the expression of Smad4 and miRNA-34a show a significant inverse correlation and the overexpression of miRNA-34a inhibits autophagy activation by directly targeting Smad4 through the TGF-β/Smad4 pathway (Sun et al., 2017).

The high mobility group box 1 (HMGB1) is an ubiquitous nuclear protein that regulates several DNA-related activities such as transcription, replication, recombination, and repair (Liu et al., 2014). HMGB1 is overexpressed in different tumor types and has been proposed as a target for cancer therapy (Lugrin et al., 2014). Among its multiple functions supporting tumor proliferation, HMGB1 improves chemotherapy resistance through the induction of autophagy in human myeloid leukemia cells (Liu et al., 2011). Interestingly, in acute myeloid leukemia (Liu et al., 2017) and in retinoblastoma cells (Liu et al., 2014), the induction of HMGB1 parallels a decreased expression of miRNA-34a, which targets HMGB1 mRNA, leading to reduced apoptosis and induction of autophagy.

As described previously, the ATG protein family is composed of 35 autophagy-related (Atg) genes: Atg1-10, 12-14, 16-18, 29, and 31 are essential for the formation of autophagosomes (Mizushima et al., 2011). Among these, Atg9 has attracted much attention because it is the only transmembrane protein among the core Atg proteins required for autophagosome formation. Fan et al. have shown that the inhibition of miR-34a restored the expression of Atg9a and significantly decreased ethanol-induced inhibition of autophagy and neural differentiation of neural crest cells (Fan et al., 2019). Huang et al. showed that miR-34a also modulates the expression of Atg9a during myocardial hypertrophy (Huang et al., 2014). These studies offer the cue to further investigations directed to the exploration of Atg9a mRNA regulation by miRNA-34a also in cancer cells.

## Delivery of Autophagy-Related miRNA-34a with Nanoparticles

As described before, the ability of miRNA-34a to target autophagy-related genes, such as *ATG4* (Wu et al., 2017)*, ATG5* (Cheng et al., 2019), *ATG9* (Yang et al., 2013), and *HMGB1* (Liu et al., 2014; Liu et al., 2017) has attracted considerable interest as a novel tool for anticancer therapy.

Therefore, the restoration of miRNA-34a physiological levels is now a promising tool to counteract tumor progression (Farooqi et al., 2017).

However, miRNAs share some properties, such as rapid biodegradation and short half-life in systemic circulation, poor biocompatibility, low membrane penetrability, and excessive off-target accumulation, that have greatly limited their application in *in vivo* systems so far (Chen et al., 2015). Thus, due to the growing awareness regarding the potential role of miRNA-34a in cancer therapy, many efforts have been addressed to optimize strategies for the targeted delivery of this miRNA.

In this sense, nanomedicine has emerged as a promising technology allowing the accumulation of systemically administered chemotherapeutics in the tumor tissues *via* the enhanced permeability and retention effect (EPR) due to leaky tumor vasculature and poor lymphatic drainage (Wilhelm et al., 2016). Extensive research focusing on developing cancer nanomedicine has generated nanostructures capable of overcoming biological barriers and transport chemotherapeutic drugs to the targeted sites while minimizing harmful effects on healthy tissues (Jain and Stylianopoulos, 2010). Moreover, the surface of nanoparticles (NPs) can be chemically modified by conjugating functional moieties, such as nucleic acids and targeting ligands, for increasing targeted delivery to the tumor sites, maximizing chemotherapy efficacy (Jabir et al., 2012).

**Figure 3:**
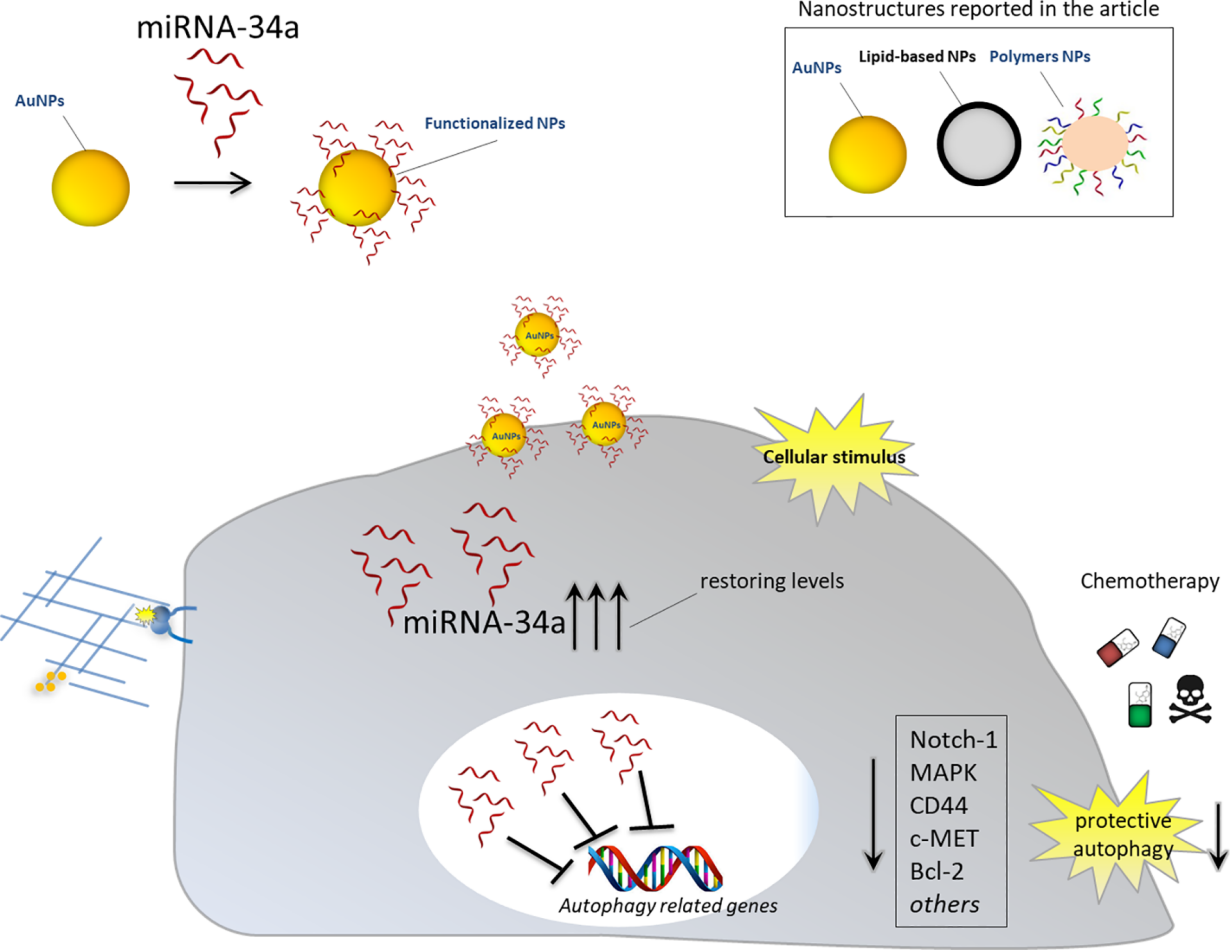
Nanoparticles for delivery of miRNA-34a for autophagy inhibition. Schematic representation showing the effects of nanoparticles functionalized with anti-autophagy miRNA-34a in cancer cells. Upon intracellular stimulus, NPs can release therapeutic miRNA-34a and restore its cellular physiological levels. miRNA-34a can exert its tumor suppressor activity by downregulating autophagy-related genes and inhibiting protective autophagy. Blocking cancer autophagy leads to cancer cell death either directly or by sensitizing them to chemotherapy treatment.

Thus, the delivery of miRNA-34a combined to nanostructures may overcome the weakness described above and function as antisense strategy to inhibit oncogenic mRNAs, involved in autophagy induction, or to restore the physiological levels of tumor suppressor miRNA-34a. Different examples regarding the employment of different nanomaterials to deliver this specific miRNA, alone or in combination with other anticancer drugs, are commented below (**Figure 3**).

## Preparation Techniques to Load miRNA in Nanoparticles

Several techniques such as emulsion based techniques (Cohen-Sela et al., 2009), nanoprecipitation (Alshamsan, 2014), interfacial polymerization (Singha et al., 2011) are used for preparation of NPs to load miRNAs. Some methods are more efficient than others based on the surface characteristics. Emulsion based nanoparticles are most commonly used methods for delivery of NPs. Emulsion based delivery method utilizes ultrasonication (Shi et al., 2014) and homogenization followed by purification at high speed centrifugation (Cosco et al., 2015). Single or double emulsion techniques are used for delivery of NPs (Jenjob et al., 2019). Oil in water emulsion is an example of single emulsion technique which can be used to encapsulate hydrophobic and hydrophilic drugs in micro- or nanoscale form. In single emulsion technique Poly(lactic-co-glycolic acid) (PLGA), one of the ideal reagent for nanoscale delivery (McCall and Sirianni, 2013), is dissolved into an organic phase (oil) that is emulsified with a surfactant or stabilizer (water). Hydrophobic drugs are added directly to the oil phase, whereas hydrophilic drugs (water) may be first emulsified with the polymer solution prior to the formation of particles (McCall and Sirianni, 2013; Jenjob et al., 2019). Double emulsion-solvent evaporation method has potential for encapsulation of both hydrophilic and hydrophobic payloads with high encapsulation efﬁciency and utilizes two emulsification steps to obtain water-in-oil-in-water (w/o/w) or oil-in water-in-oil (o/w/o) emulsions (McCall and Sirianni, 2013; Jenjob et al., 2019).

Another technique used for delivery of NPs is nanoprecipitation. It is a less complex and widely applicable method for hydrophobic drug molecules (Salatin et al., 2017). It is a solid displacement method which requires solvent (organic) and nonsolvent (inorganic) phase separation followed by addition of one phase to another (Salatin et al., 2017). Both the drug and the polymer for the delivery must be dissolved in solvent; once the solvent is mixed to a nonsolvent rapid desolvation and precipitation of polymer starts leading to drug entrapment (Fessi et al., 1989; Martínez Rivas et al., 2017). Solvents miscible with water and nonhalogenated solvents are commonly used; however, immiscible solvents such as dichloromethane can also be used (Salatin et al., 2017). One example is mixing of cucurbitacin I and PLGA in acetone. The organic phase is added dropwise to deionized water containing 1% Pluronic F-68 followed by acetone evaporation (Alshamsan, 2014). Another example in which miRNA-loaded PLGA/chitosan (PLGA/CS) NPs of 150–180 nm size are prepared *via* the nanoprecipitation method is by dropwise addition of PLGA solution into a water solution of CS and miR-34s in the presence of a Poloxamer surfactant (Salatin et al., 2017).

Interfacial polymerization is another technique used to deliver NPs. Using this approach hydrophilic substances such as miRNAs are incorporated into biodegradable lignin nanocontainers (Wurm and Weiss, 2014). This technique involves copolymerization of hydrophobic and hydrophilic monomers to constrain the polymerization at interfaces, but its free-radical mechanism allows precise control of initiation, which makes it possible to finely disperse the immiscible phases prior to polymerization (Scott et al., 2005). The interfacial polymerization falls into different types of interfaces: liquid–solid interfaces, liquid–liquid interfaces, and liquid-in-liquid emulsion interfaces (Song et al., 2017). There are also other interface categories, rarely used, including liquid–gas, solid–gas, and solid–solid (Salatin et al., 2017; Song et al., 2017).

### Inorganic Nanoparticles

Recent advancement in nanotechnology has led to the introduction of various inorganic nanomaterials, such as silica dioxide nanoparticles (SiO_2_-NPs), calcium phosphate nanoparticles (CaP-NPs), gold nanoparticles (AuNPs), gold nanoshells (AuNShs), and magnetic nanoparticles (MNPs) that have been exploited as excellent nanocarriers for the delivery of nucleic acids both *in vitro* and *in vivo* models (Naz et al., 2019). These nanomaterials share some properties such as high bioactivity, biocompatibility, and chemical stability that make them efficient delivery systems (Cheng and Kuhn, 2007; Ghosh et al., 2008; Park et al., 2009; Tang et al., 2012; Xu et al., 2014; Xiong et al., 2018). Recently, it has been reported that SiO_2_-NPs can efficiently deliver active miR-34a into breast cancer cells leading to the reduction in mammosphere formation (Panebianco et al., 2019) and the downregulation of Notch-1, a well-established miR-34a target with an important role in regulating stem cell functions and autophagy process (Bouras et al., 2008; Marcel and Sarin, 2016). Notably, *in vivo* miR-34a delivery by SiO_2_-NPs was able to exert a tumor-suppressive function leading to the reduction of breast cancer in engrafted mice (Panebianco et al., 2019) (**Table 1**).

**Table 1:** Nanoparticles for delivery of autophagy-related miRNA-34a as cancer treatment.

Entry	Nanoparticles	Compound carried/co-treatment	Tested cell lines (tissue of origin)	*In vivo* models	Biological effects	Molecular mechanisms	Refs
1	SiO2-NPs	None	SUM159pt; mammospheres (breast cancer)	mutant miR34-TKO mice; NOD.Cg-Prkdc^scid^Il2rg^tm1Wjl^/SzJ mice	Reduction of mammosphere formation; Reduction in tumor growth *in vivo*.	Notch-1↓	(Panebianco et al., 2019)
2	CaPs	Linear polyethyleneimine (LPEI)	PC3 (prostate cancer)	None	Inhibition of cell proliferation; Inhibition of cell migration	Not reported	(Jung et al., 2015)
3	AuNPs	7-ethyl-10-hidroxycamptothecin (SN-38) microRNA-182, microRNA-137 and microRNA-144	Mel-202 (uveal melanoma)	none	Synergistic increase in cytotoxicity; Sensitization to SN-38	c-MET↓	(Milán Rois et al., 2018)
4	AuNSsh	None	MDA-MB-231 (breast cancer)	None	Inhibition of cell proliferation; Inhibition of metabolic activity	SIRT1↓; Bcl2↓	(Li et al., 2013)
5	MNPs	Nitrosonium tetrafluoroborate (NOBF4); Polyethylene glycol (PEG)	Not reported	None	Inhibition of cell migration and invasion	CD44↓	(Lee et al., 2016)
6	CPP-9 (CRGDKGPDC)	b-cyclodextrin-polyethylenimine (PC)	PANC-1 (pancreatic cancer)	Athymic female mice (BALB/c strain)	Cell cycle arrest; apoptosis; suppression tumor cell migration; reduction tumor growth *in vivo*.	E2F3↓; Bcl-2↓; c-myc↓; cyclin D1↓	(Hu et al., 2013)
7	CPP Nona arginine (R9)	c(RGDfK) and Folic acid (targeting ligands)	U87MG and HeLa (Breast and ovarian cancer)	None	Not reported	Not reported	(Xiao et al., 2018)
8	SLNs	Dimethyldioctadecylammonium bromide (DDAB)	B16F10-CD44+ (Lung metastasis of melanoma)	Female C57BL/6 mice	Induction of apoptosis; Reduction of cell migration; Reduction of spheroids formation *in vitro*; Inhibition of tumor growth and metastasis *in vivo*	CD44↓	(Shi et al., 2013)
9	SLNs	Paclitaxel (PTX)	B16F10-CD44+ (Lung metastasis of melanoma)	Female C57BL/6 mice	Synergistic inhibition of cancer development and metastasis	CD44↓	(Shi et al., 2014)
10	LPH	Polyethylene glycol (PEG), Single-chain antibody fragment (scFv)	B16F10-CD44+ (Lung metastasis of melanoma)	None	Induction of apoptosis	Survivin↓ MAPK↓	(Chen et al., 2010)
11	PAMAMs	Phenylboronic acid (PBA)	BGC-823 (Gastric cancer)	BALB/c-nude mice	Induction of apoptosis; Reduction of cancer cells invasion and migration; Reduction of tumor growth *in vivo*.	Notch-1↓	(Song et al., 2019)
12	PAMAMs	S6 aptamer	A549 (Lung cancer)	None	Induction of apoptosis; Inhibition of cell growth, cell migration and invasion	P53↓ Bcl-2↓	(Wang et al., 2015a)
13	DSS-BEN	1,N11-bisethylnorspermine (BENSpm).	HCT116 (colorectal carcinoma)	Xenograft athymic nude mice	Inhibition of polyamines metabolism; Cancer cell death; Inhibition of tumor growth *in vivo.*	SMOX↑ SSAT↑ Bcl-2↓	(Xie et al., 2017)
14	CBSA	Docetaxel (DTX)	4T1, A549 (breast and lung cancer)	4T1 tumor-bearing mice	Induction of apoptosis; Cytotoxicity; Inhibition of cell migration; Inhibition of tumor growth *in vivo.*	Bcl-2↓	(Zhang et al., 2017)
15	HP-IPECs	None	MDAMB-231 and MCF-7 (breast cancer)	Female athymic nude Balb/c mice	Induction of apoptosis; Inhibition of cell migration; inhibition of cell proliferation; reduction of tumor growth *in vivo*.	Notch-1↓ CD44↓	(Wang et al., 2015b)
16	Chitosan-NPs	Poly(D,L-lactide-co-glycolide) (PLGA)	SKMM1 and RPMI8226 (multiple myeloma)	Immunodeficient NOD–SCID mice	Reduction cell proliferation and toxicity in cell lines; Reduction of tumor growth *in vivo*.	Bcl-2↓ CDK6↓	(Cosco et al., 2015)
17	Chitosan-NPs	Hyaluronic Acid (HA) Doxorubicin (DOX)	MDA-MB-231 (triple negative breast cancer)	Female athymic nude Balb/c	Induction of apoptosis; Reduction of cell proliferation; Inhibition of cancer cell migration; Inhibition of tumor growth *in vivo*.	Bcl-2↓ Notch-1↓	(Deng et al., 2014)
18	Hyaluronic acid-poly(ethylene imine) (HA-PEI)-NPs HA-poly(ethylene glycol) (HA-PEG)	None	A549 (cisplatin sensitive) and A549 DDP (cisplatin resistant) (lung cancer)	None	Increased ATP levels; Reduction glycolysis; Elevation of mitochondrial complexes activity; Gene-Specific Changes in mtDNA encoded genes; Alteration of mitochondrial epigenetic enzymes; Induction of apoptosis.	Bax↑ Apaf-1↑ PUMA↑ caspase3↑ Bcl-2↓ survivin↓ mt-TET1-3↑ Complex I ↑ Complex IV↑	(Trivedi et al., 2017)
19	β- cyclodextrin-PEI600-NPs	polyethylene glycol (PEG)	B16F10 (melanoma)	B16F10-CD44+ xenograft model of female C57BL/6 mice	Inhibition of tumor progression; Inhibition of cell invasion; Inhibition of sphere formation; Reduction of tumor growth *in vivo.*	CD44↓	(Fan et al., 2017)
20	poly(L-lysine-graft-imidazole) (PLI)	polyethylene glycol (PEG)	MKN-74 (gastric cancer)	Orthotopic and subcutaneous xenograft model of male BALB/c-nude mice	Reduction metastasis and cell invasion; Induction of apoptosis; Inhibition of tumor relapse and growth *in vivo.*	CD44↓ Bcl-2↓ Oct-3/4↓ Nanog↓	(Jang et al., 2016)

In a study performed by Mok et al., a long chain miRNA-34a (lc-miRNA-34a) was prepared by chemical crosslinking in order to improve encapsulation efficiency into linear polyethyleneimine (LPEI)-coated CaP-NPs (LPEI-CaP) and intracellular delivery (Jung et al., 2015). These nanoformulations were successfully delivered into PC-3 cancer cells, where miRNA-34a was efficiently released, suppressing cancer cell proliferation as well as cell migration (Jung et al., 2015).

In another study by Milán-Rois et al., a therapeutic mixture containing SN-38(7-ethyl-10-hidroxycamptothecin), a topoisomerase inhibitor (Palakurthi, 2015), and miRNA-34a has been delivered in uveal melanoma cells using AuNPs (Milán Rois et al., 2018). The nanoformulations led a synergistic cytotoxicity effect in cancer cells and were able to reprogram their oncogenic phenotype by downregulating c-MET tyrosine kinase, making Mel-202 cancer cells more susceptible to SN-38 (Milán Rois et al., 2018).

Other inorganic nanostructures widely used in biomedical applications are gold nanoshells (AuNShs), which are composed of an inorganic core coated with a thin layer of gold (Bardhan et al., 2011).

In a recent study, Goyal et al. reported that layer-by-layer assembled gold nanoshells (LbL-AuNShs) were able to efficiently deliver miRNA-34a to MDA-MB-231 breast cancer cells and release it upon intracellular stimulus (Goyal et al., 2018). Notably, the cancer cells treated with these inorganic nanostructures downregulated the expression of the autophagy-modulators SIRT1 and Bcl2, which are known downstream targets of miRNA-34a (Li et al., 2013). Moreover, treatment with LbL-AuNSsh affected the proliferation and metabolic activity of triple-negative breast cancer cells without negatively impacting noncancerous MCF10A breast epithelial cells (Li et al., 2013).

MNPs are ideal candidates for surface modifications generating functional nanostructures that can be employed in a variety of biomedical applications, including drug delivery for cancer therapy (Xiong et al., 2018). In this regard, MNPs modified with nitrosonium tetrafluoroborate (NOBF4) and polyethylene glycol (PEG) have been successfully employed for the delivery of miR-34a in cancer cells. The miRNA-34a conjugated nanostructures led to the downregulation of CD44 (Lee et al., 2016), a protein upregulated by autophagy and involved in chemoresistance in oral squamous cell carcinoma (Naik et al., 2018). The inhibition of CD44 expression showed therapeutic effects, such as the reduction of cell migration and invasion, suggesting that magnetic nanostructures have the potential for miRNA-based cancer therapy.

### Lipid-Based Nanoparticles

Among the many lipid-based nanocarriers that have been developed during the last years, solid lipid nanoparticles (SLNs) have attracted considerable interest for drug delivery and targeting (Mishra et al., 2018). SLNs can include a cationic component that facilitates association with anionic miRNAs and are able to protect them from degradation during systemic circulation (Müller et al., 2000). Interestingly, Shy et al. developed a lipid nanoparticle system containing SLNs and cationic dimethyl dioctadecyl ammonium bromide (DDAB) to carry miRNA-34a in cancerous lung for CSC therapy (Shi et al., 2013). These miRNA-34a-SLNP nanocomplexes induced B16F10-CD44+ cell apoptosis and inhibited cell migration by negatively regulating the cell surface protein CD44.

Moreover, the nanoformulations were also effective in inhibiting B16F10-CD44+ tumor development and tumorigenicity *in vivo* (Shi et al., 2013).

However, when miRNA-34a and paclitaxel (PTX), an inhibitor of microtubules dynamic (Jordan and Wilson, 2004), were coincorporated into SLNs, the combination of these two drugs could cooperatively and more efficiently induce cell apoptosis and inhibit B16F10-CD44+ development in *in vitro* and *in vivo* models through different mechanisms (Shi et al., 2014).

Apart from SLNs, it has been shown that liposome-polycation-hyaluronic acid (LPH) nanoparticles could systemically deliver therapeutic siRNA into the tumor site with relatively low toxicity (Li et al., 2008). In this regard, Huang et al. employed LPH nanoparticles modified with PEG and tumor-targeting single-chain antibody fragment (scFv) for systemic delivery of the anti-autophagic miRNA-34a in experimental lung metastasis of murine B16F10 melanoma (Chen et al., 2010). Interestingly, miRNA-34a was found to inactivate the MAPK pathway, leading to therapeutic activity in B16F10 melanoma cells (Chen et al., 2010). MAPK pathway has been shown to induce autophagy through phosphorylation of c-Jun/c-Fos transcription factors, which in turn lead transcription of autophagy-related genes (Zhou et al., 2015; Lee et al., 2019).

### Polymeric Nanoparticles

Amine terminated polyamidoamines (PAMAMs) are a class of synthetic polymers that have been used as nanocarriers because of their unique properties, such as highly branched structure, water-solubility, high charge density, and numerous amine groups for further modification (Han et al., 2018). Besides, conjugation with specific tumor-targeted ligands is an effective way for facilitating the intracellular delivery of PAMAMs in a receptor-mediated endocytosis manner (Chen et al., 2017). Phenylboronic acid (PBA) exhibits a high affinity with sialic acid (SA), which is overexpressed in various types of tumor cells (Narayanan, 1994), making it a promising tumor-targeted ligand (Jia et al., 2014).

In a recent work, PBA was successfully attached to a PAMAM surface to obtain a functional PAMAM (PPP), which has been employed for miRNA-34a delivery in gastric carcinoma cell line BGC-823 (Song et al., 2019). Interestingly, PPP/miRNA-34a nanocomplexes were found to counteract the autophagic Notch-1 signaling pathway resulting in the induction of apoptosis and the inhibition of cell migration and invasion (Song et al., 2019).

Alternatively, PAMAM may be conjugated with aptamers to generate nanocomplexes, which display several properties, such as high affinity and specificity to the target molecules, less toxicity, and rapid tissue penetration (Tan et al., 2011). In this regard, miRNA-34a has been encapsulated into S6 aptamer conjugated dendrimer to form lung cancer-targeted delivery nanoparticles (PAM-Ap/pMiRNA-34a NPs) (Wang et al., 2015a). The aptamer conjugation improved cellular uptake of miRNA-34, which targeted important genes involved in autophagy regulation, such as Bcl-2 and p53 (Lindqvist et al., 2014; Mrakovcic and Fröhlich, 2018). Thus, PAM-Ap/pMiRNA-34a-NPs showed therapeutic activity by inhibiting cell growth, migration, invasion and by inducing apoptosis of lung cancer cells (Wang et al., 2015a).

These studies provide novel therapeutic strategies, based on dendrimer nanoparticles, to deliver tumor suppressor miRNA-34 in cancer cells to target autophagy-related mechanisms and counteract tumor progression and cancer growth.

Natural polyamines, such as spermidine and spermine, are key regulators of cell growth, differentiation, and survival (Pegg and Casero, 2011). Recently, spermidine was found to induce autophagy by inhibiting the lysine acetyl transferase E1A-binding protein p300 (EP300) (Pietrocola et al., 2015). In cancer, dysregulation of polyamine metabolism promotes tumor development and progression (Lu et al., 2011) and represents a promising target in cancer chemotherapy (Basuroy and Gerner, 2006).

In this sense, in a study performed by Oupický et al., biodegradables nanoparticles synthesized from a polyamine analog N1, N11-bisethylnorspermine (BENSpm) were employed to efficiently deliver miR-34a in colorectal cancer cells (Xie et al., 2017). After treatment with an intracellular stimulus, these nanostructures could disassemble in the cytosol releasing both the miRNA-34a and BENSpm, which exerted a therapeutic function by downregulating Bcl-2 and inducing the expression of enzymes involved in polyamine catabolism, such as SMOX and SSAT (Stewart et al., 2018).

The ability of these biodegradable nanocarriers to deliver therapeutic miRNA-34a and inhibit polyamine metabolism may provide an efficient approach to combination nanomedicines for autophagy inhibition in cancer therapy.

Recent studies have evidenced that self-assembly strategies are useful tools to generate various nanomaterials for drug delivery (Declerck et al., 1990; Naito et al., 2012). In this regard, interpolyelectrolyte complexes (IPECs) can be formed by self-assembly mixing two oppositely charged polyelectrolytes in aqueous solution (Pergushov et al., 2012). By using the IPEC-based approach, nucleic acids have been efficiently absorbed on the cationic surface of the nanocomplexes and successfully delivered in cells (Cheng et al., 2009; Kim et al., 2009).

In a work by Wang et al, miRNA-34a has been delivered into breast cancer cells using nanocapsules prepared through IPECs-based approach and composed of cationic protamine sulfate (PS) and anionic Hyaluronic Acid (HA) (Wang et al., 2015b). Notably, nanocomplex-assisted delivery of miRNA-34a induced apoptosis and suppressed migration and proliferation of breast cancer cells as well as reduced tumor growth in a xenograft mouse model *via* targeting signaling pathways autophagy-related, as CD44 and Notch-1 (Wang et al., 2015b). Therefore, this biodegradable nanoplatform provides a great potential for miRNA-34a based therapy against triple-negative breast cancer.

Amiji et al. developed a novel drug delivery system incorporating self-assembling hyaluronic acid-poly(ethylene imine) (HA-PEI) and HA-poly(ethylene glycol) (HA-PEG) nanoparticles to efficiently deliver tumor suppressor miR-34a inA549 lung cancer cells for redox-epigenetic modifications (Ganesh et al., 2013; Trivedi et al., 2017). The authors reported that miRNA-34a HA-NPs treatment resulted in decreased glycolytic flux and antioxidant response element Nrf-2 resulting in depleted glutathione levels and ultimately, increased in several pro-apoptotic factors in both cisplatin-sensitive and cisplatin-resistant lung adenocarcinoma cells (Trivedi et al., 2017). Remarkably, molecular changes in epigenetic status on both mitochondrial (mt) and nuclear (nc)DNA and transcription of mtDNA-encoded genes were also observed after treatment with miRNA-34aHA-NPs. Interestingly, some studies report the existence of a functional interplay between glycolytic metabolism and autophagy in a positive loop that sustains tumor progression (Watson et al., 2015; Fan et al., 2018; Jiao et al., 2018). Therefore, the delivery of miRNA-34a through HA-PEI/HA-PEG nanoparticles may represent a novel challenge to counteract the Warburg effect and autophagy of cancer cells by promoting a glycolytic to oxidative metabolism switch and inducing deep changes in their metabolic settings, correlating with alterations of cell proliferation.

Other studies reported that polymeric nanostructures composed of biocompatible cationic *β*-cyclodextrin-PEI600 (CDP) or poly(L-lysine-graft-imidazole) (PLI) were loaded with anionic miRNA-34a and subsequently PEGylated to protect them from degradation, thus allowing a safe and efficient intracellular delivery (Jang et al., 2016; Fan et al., 2017).

These pH-sensitive nanoparticles released therapeutic miRNAs in the acid tumor microenvironment, favoring its cytoplasmic uptake by gastric and melanoma cancer cells.

The miRNA-34 loaded NPs repressed the expression of oncogenic CD44 protein with decreased levels of Bcl-2, Oct-3/4 and Nanog genes, thus leading the suppression of CSCs-like characteristics, induction of apoptosis, reduction of cell invasion and metastasis and tumor growth inhibition in xenograft gastric cancer models (Jang et al., 2016; Fan et al., 2017).

These polymer-based strategies of miRNA-34a delivery might represent novel therapeutic challenges with highly selective tumor cell death and tumor growth inhibition in CD44-positive tumors, thus opening a therapeutic window for autophagy inhibition for cancer treatment.

### Biopolymer-Based Nanoparticles

Nanomaterials based on bovine serum albumin (BSA) are nontoxic, nonantigenic, and biodegradable polymers and have been widely exploited in drug delivery (Rhaese et al., 2003). The anionic side-chain carboxylic groups can be modified with a cationic amino group to obtain cationic bovine serum albumin (CBSA) (Fischer et al., 2001). This cationic structure can be conjugated to nanoparticles and act as a safe drug delivery system for nucleic acids (Han et al., 2014).

In this regard, core–shell nanocarriers coated by CBSA were developed for delivery of miRNA-34a and the chemotherapeutic drug docetaxel (DTX) for a cosynergistic treatment of metastatic breast cancer (Zhang et al., 2017). The coloaded nanocarriers (CNCs) could internalize through the caveolae-mediated pathway and exerted therapeutic effect by inducing cytotoxicity *in vitro* and by inhibiting tumor growth and metastasis in 4T1-tumor-bearing mice models (Zhang et al., 2017). Thus, these nanocarriers represent new nanoplatforms for the delivery of therapeutic miRNA-34 and provide a promising strategy for the treatment of metastatic breast cancer.

Chitosan, a partially deacetylated derivative of chitin composed of N-acetylglucosamine, has emerged as significant biopolymer for drug delivery because of its unique chemical proprieties such as biocompatibility, biodegradability, low toxicity, and easy modification (Mohammed et al., 2017). In a study performed by Cosco et al., safe chitosan/PLGA nanocomplexes have been developed that were able to efficiently encapsulate and deliver miRNA-34a in order to provide a new tool for the treatment of multiple myeloma (Cosco et al., 2015). These nanostructures led to a significant *in vitro* antitumor effect by reducing the proliferative capabilities of multiple myeloma cells. Moreover, the systemic injection of miRNA-34a-loaded nanoparticles significantly inhibited tumor growth through the downregulation of Bcl-2 and CDK6 expression and improved the survival of multiple myeloma xenografts in NOD-SCID mice (Cosco et al., 2015).

Among the various polymeric nanocomplex systems, hyaluronic acid-chitosan nanoparticles (HA-CS NPs) have been extensively studied. In addition to its biocompatibility and biodegradability, HA backbone possesses tumor-targeting properties through specific binding to CD44, an integral membrane glycoprotein overexpressed on the surface of various tumor cells (Aruffo et al., 1990) that makes it as an ideal polymer carrier for systemic drug delivery applications (De La Fuente et al., 2008).

It has been reported that HA-CS nano-complexes were able to simultaneously encapsulate and deliver positively charged Doxorubicin (DOX) and negatively charged miRNA-34a mimics into triple-negative breast cancer cells for improved chemotherapeutic effects (Deng et al., 2014). Interestingly, through the restoration of endogenous miRNA-34a levels, these nanoformulations synergistically enhanced antitumor effects of DOX in both *in vitro* and *in vivo* models by suppressing the expression of nonpump resistance and anti-apoptotic Bcl-2 protein. In addition, the delivery of miRNA-34a inhibited breast cancer cell migration *via* targeting Notch-1 signaling (Deng et al., 2014). Hence, nanosystem-based codelivery of chemotherapeutic agents and tumor suppressor miRNA-34a may be a promising combined therapeutic strategy for enhanced antitumor therapy.

## Conclusions

The discovery and characterization of noncoding RNAs in the last 20 years have opened a new layer in the understanding of gene expression physiology as well as novel therapeutic strategies. In particular, various miRNAs, and miRNA-34a in particular, have been characterized as powerful tools regulating the expression of key genes relevant in the development of a disease, such as the control of autophagy during carcinogenesis.

However, despite many years of intense study, the translational impact on this knowledge is not satisfactory. Specific biologic characteristic of miRNAs, such as poor chemical stability and low membrane permeability, may preclude their use in clinics. For this purpose, nanotechnology has developed a bunch of new approaches aimed at enhancing the *in vivo* stability and at facilitating the delivery of specific miRNAs to the site of disease.

Thus, the use of several nanomaterials to deliver therapeutic nucleic acids, such as miRNA-34a, in cancer cells may result in the inhibition of protective autophagy leading to a tumor suppressor phenotype such as apoptosis induction, increased chemosensitivity and therapeutic efficacy.

The high potentiality of the use of encapsulated miRNAs is supported by the generation of MRX34, a lipid nanoparticle filled with miR-34 mimics, that has been the first microRNA-associated therapeutic drug tested in a clinical trial (Zhang et al., 2019). Indeed, a study on adults affected by solid tumors refractory to standard treatment that have been treated twice weekly for three weeks in 4-week cycles with MRX34 showed that this formulation exerts an evident antitumoral activity (Beg et al., 2017). Interestingly, the administered MRX34 was also found to be present in various tissues, including liver, bone marrow, spleen, mammary gland, and lung (Kelnar and Bader, 2015), thus supporting its application in the treatment of numerous cancer types.

Another evidence of the therapeutic application of delivered miRNA has been proposed by the use of the coencapsulated miR-34a and let-7b to NSCLC mice resistant to conventional anticancer therapy. The obtained data showed that the dual treated animals have a reduced tumor burden and a prolonged survival (Kasinski et al., 2015). Taking into account these data and the here reported formulations and applications of miRNA-34a delivery, the therapeutic usefulness of the development of novel miR-34a formulations may help to successfully achieve the clinical trial.

However, despite the potential advantages of the described nanoparticles, it is necessary to remember that the use of these nanomedicines may display some toxicity. Indeed, in some cases, nanomaterials may overstimulate autophagy in healthy tissues leading to dangerous effects, including inflammation, oxidative stress, and neoplastic transformation (Cordani and Somoza, 2019). Hence, it should be kept in mind that such nanostructures can produce undesired side effects whereby even if miRNA-34a is delivered in cancer cells, autophagy might increase in the same way by eliminating the beneficial effect of that therapeutic molecule.

In conclusion, deeper integration of knowledge on the role of autophagy in tumors with the recent advances in nanomedicine may allow an effective use of microRNA in the therapy of tumors.

## Author Contributions

All authors contributed to the article and approved the submitted version. MC conceived the article, supervised the study, and coordinated the writing of the manuscript.

## Funding

This work was supported by the Spanish Ministry of Economy and Competitiveness (SAF2017-87305-R) and by Fondi di Ateneo 2019 (Sapienza University). IMDEA Nanociencia acknowledges support from the ‘Severo Ochoa’ Programme for Centres of Excellence in R&D (MINECO, Grant SEV-2016-0686).

## Conflict of Interest

The authors declare that the research was conducted in the absence of any commercial or financial relationships that could be construed as a potential conflict of interest.
